# Case report: low-titre anti-Yo reactivity in a female patient with psychotic syndrome and frontoparieto-cerebellar atrophy

**DOI:** 10.1186/s12888-015-0486-x

**Published:** 2015-05-12

**Authors:** Dominique Endres, Evgeniy Perlov, Oliver Stich, Philipp Tobias Meyer, Niklas Lützen, Ludger Tebartz van Elst

**Affiliations:** 1 Section for Experimental Neuropsychiatry, Department of Psychiatry and Psychotherapy, University Medical Center Freiburg, Hauptstr. 5, 79104 Freiburg, Germany; 2 Department of Neurology, University Medical Center Freiburg, Breisacher Str. 64, 79106 Freiburg, Germany; 3 Department of Nuclear Medicine, University Medical Center Freiburg, Hugstettet Str. 64, 79106 Freiburg, Germany; 4 Department of Neuroradiology, University Medical Center Freiburg, Breisacher Str. 64, 79106 Freiburg, Germany

**Keywords:** Anti-Yo reactivity, Low titre, Cerebellum, Atrophy, Paraneoplastic neurological syndrome

## Abstract

**Background:**

Autoimmune and inflammatory mechanisms in psychotic disorders have attracted increasing scientific attention in recent years. In this regard, we performed routine cerebrospinal fluid (CSF) basic diagnostics and CSF/serum analyses for antibodies directed against neuronal intracellular and surface antigens in psychotic patients. In this context, the patient presented in this paper was diagnosed.

**Case presentation:**

We present the case of a 20-year-old female patient with a first episode of a drug-induced psychotic syndrome but without neurological deficits. Further investigations showed a reproducible low-titre positive anti-Yo reactivity in the CSF and serum with two independent immunoblot assays. Magnetic resonance imaging showed frontoparietal and cerebellar atrophy. On [^18^F]fluorodeoxyglucose positron emission tomography, a mild cerebellar hypometabolism was found. No underlying tumor was detected.

**Conclusion:**

Despite the presence of anti-Yo reactivity, the diagnostic criteria for a paraneoplastic neurological syndrome were not fulfilled. Previously published data indicate the possible association between low-titer antibodies against intracellular localized, onconeural antigens, and psychotic disorders. Large prospective studies that investigate the prevalence and clinical significance of antibodies against intracellular onconeural antigens in psychiatry are needed.

## Background

In recent years, discrete immunological encephalopathy (IE) has been increasingly recognized as a possible cause of psychotic and affective disorders [1,2]. In the majority of cases, other more or less subtle findings, such as magnetic resonance imaging (MRI), electroencephalography (EEG) pathology, or neurological soft signs point to an “organic cause” of the psychotic syndrome; however, a number of case reports in the literature indicate that IE is identified in psychotic cases without these findings [3] (Endres D, Perlov E, Stich O, Tebartz van Elst L: Steroid responsive encephalopathy associated with autoimmune thyroiditis (SREAT) - presenting as major depression, in preparation). For this reason, in our institution, we have been offering serum and cerebrospinal fluid (CSF) analyses for antibodies directed against neuronal intracellular and surface antigens as well as CSF basic diagnostics as a routine diagnostic measure to all psychotic patients since 2009. In this paper, we report the case of a schizoaffective patient with low-titer anti-Yo reactivity in the CSF and serum, respectively, whom we have identified in this context. Anti-Yo antibodies belong to the so-called “well-characterized” paraneoplastic antibodies against intracellular onconeuronal antigens [4]. They target the Purkinje cells of the cerebellum and are therefore called the Purkinje cell cytoplasmic antibodies type 1 [5]. The anti-Yo syndrome in association with high-titer anti-Yo antibodies is the most common reason for paraneoplastic cerebellar degeneration (PCD). In PCD, global cerebellar deficits typically arise sub-acutely and progress over months. Disturbances in cognitive impairment with memory deficits and emotional instability are also described [6-8]. The anti-Yo syndrome is primarily associated with gynecological (typically ovarian but also breast, uterine, or tubal) carcinomas [8].

## Case presentation

### Clinical presentation

We present the case of a 20-year-old female high school graduate with an acute schizoaffective syndrome. She complained of auditory (contrasting voices) and visual (images on the sky or migratory objects) hallucinations. She also experienced a sensation as if the wind was blowing into her face (delusional atmosphere), and she believed this to be a sign from her unfamiliar environment (delusion of influence). She interpreted these signs as a call for suicide. Moreover, she reported depressive symptoms, such as sadness, loss of motivation and energy, social withdrawal, insomnia, and lack of appetite. Her capability to think or concentrate adequately was reduced, and her overall thinking processes were slow. After being admitted to the hospital, she also complained of a headache. Comprehensive medical and neurological examinations indicated no relevant abnormalities, with the exception of a mild dysdiadochokinesia and a moderate hirsutism.

### Previous medical history

This was the first episode of psychiatric symptoms for the patient. She had no history of complications during pregnancy or delivery, and early development was unremarkable and without any evidence of a neurodevelopmental disorder. She was socially integrated and successfully completed her academic career. However, the patient reported a diffuse episode of earlier intermediate gait disorder resembling ataxia. The onset of symptoms was temporally related to short-term consumption of hallucinogenic mushrooms and rare but repeated low-dose cannabis abuse. The psychotic symptoms developed over a subsequent five-month period prior to admission to our hospital despite drug abstinence. No tumor in the patient’s own history was known, but a family history of breast cancer was positive (grandmother: breast cancer at age of 30 years).

### Diagnostic results

**Figure 1:**
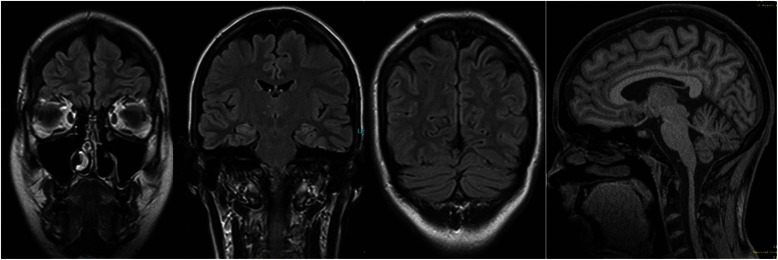
Magnetic resonance imaging shows a slight but atypical age-related frontoparietal and cerebellar atrophy.

**Figure 2:**
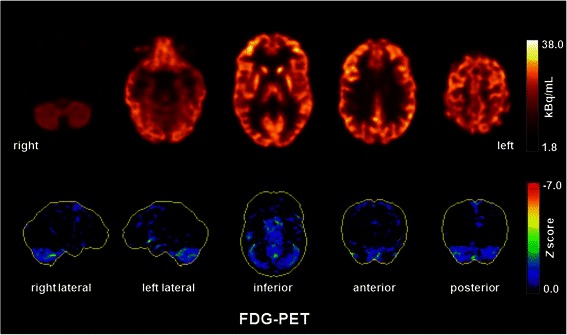
[^18^F]fluorodeoxyglucose positron emission tomography (FDG-PET) showed a mild, borderline significant cerebellar hypometabolism. Otherwise, the PET was unremarkable. No mesiotemporal hypermetabolism as typically seen in limbic encephalitis was observed. FDG-PET was performed at the Department of Nuclear Medicine of the University Hospital Freiburg after injection of 320 MBq FDG (Gemini True Flight, Philips Electronics, The Netherlands). Upper and lower row images show transaxial FDG-PET images and 3D surface projections of regions with decreased FDG uptake (colour-coded *Z*-score, compared to age-matched healthy controls), respectively.

Serum and CSF investigations with the “ravo blot” using recombinant onconeural antigens as a substrate showed a weak but reproducible anti-Yo specific reactivity [9] (ravo blot: immunoblot using recombinant onconeural antigens as a substrate; ravo Diagnostika, Freiburg, Germany [www.ravo.de/de/Produkte/ravo_pns_blot_zu_immuno_verst.php]) but no evidence of an anti-Yo specific intrathecal antibody synthesis. In the Euroimmun immunblot for serum and CSF an anti-Yo specific, reactivity was found too. Moreover, a weak anti-Ma2-reactivity was found in the serum (without confirmation in the ravo blot; Euroimmun blot: EUROLINE –Neuronal Antigens Profile 4, Euroimmun, Luebeck, Germany [www.euroimmun.de/index.php?id=29&L=1]). No antibodies were found against other intracellular onconeural antigens (Hu, CV2/CRMP5, Ri, Ma1, SOX1) or intracellular synaptic antigens (GAD, amphiphysin) in the serum. Immunological screening in the serum for rheumatoid autoimmune disorders was negative. In addition, serological analysis showed no exposure to neurotropic infectious agents, such as borrelia, lues, HIV, and FSME. CSF analysis was unremarkable (white blood cell count: 1/μL, no dysfunction of the lood-brain-barrier [albumin-quotient: 4.1], no intrathecal IgG synthesis). Moreover, no antibodies against neuronal cell surface antigens (NMDAR, AMPA-1/2-R, GABA-B-R, VGKC-complex [LGI1, Caspr2]) were found in the CSF. EEG was normal, whereas MRI illustrated a discrete frontoparietal and cerebellar atrophy, which was clearly abnormal given the young age of the patient (Figure 1). In addition, a small arachnoidal cyst was detected in the left temporopolar region. The arachnoidal cyst is an incidental finding without a substantial space-occupying effect; therefore, it is not very likely that it leads to clinical symptoms. [^18^F]fluorodeoxyglucose positron emission tomography (FDG-PET) depicted a mild, borderline significant cerebellar hypometabolism. Otherwise, the PET was unremarkable. In particular, mesiotemporal hypermetabolism, which is typically seen in limbic encephalitis, was not observed (Figure 2). The broad diagnostic work-up in search for primary tumors remained unremarkable (breast ultrasonography, whole-body FDG-PET/computed tomography, chest MRI). However, a polycystic ovary syndrome (with secondary amenorrhea, polycystic ovaries, and hirsutism) was diagnosed.

### Therapeutic and clinical course

On a clinical basis, we started a combined therapy with risperidon (4mg/day) and quetiapine (300mg/day). Psychotic symptoms declined under medical treatment after one to two weeks. Depressive symptoms, such as loss of motivation or energy and a diminished ability to think or concentrate, deteriorated. Interestingly, anti-Yo reactivity in the serum disappeared four months after the first positive result without any immunosuppressive treatment.

## Conclusions

### Characteristics of our case report

We present the case of a 20-year-old woman suffering from a schizoaffective syndrome. Laboratory diagnostics showed a weak and transient anti-Yo-specific reactivity in the CSF and serum, respectively. This abnormal laboratory finding was confirmed by examination of consecutive samples with two independent immunoblot assays using highly specific and recombinant onconeural antigens that showed reproducible anti-Yo reactivity until its spontaneous disappearance after fourth months. MRI depicted a slight but clearly abnormal frontoparieto-cerebellar atrophy, and FDG-PET detected a hypometabolism of both cerebellar hemispheres. While assuming that the psychiatric symptoms were triggered by the transient drug abuse is plausible, according to present knowledge and thinking, it does not explain the MRI and FDG-PET findings nor the anti-Yo reactivity.

### Diagnostic assessment and differential diagnosis

Paraneoplastic neurological syndromes (PNS) are characterized by high and permanent concentrations of antineuronal antibodies and are frequently associated with intrathecal antigen-specific antibody synthesis, inflammatory CSF changes, distinct neurological syndromes, and, very often but not always, the detection of cancer during further diagnostic work-up [8,10]. PCD is a well-established PNS. Our patient here showed suspicious findings in the cerebral MRI and FDG-PET for this syndrome, although additional frontoparietal atrophy is uncommon. At the time of imaging acquisition, no clinical cerebellar signs were observed, and the patient’s history indicated only one transient episode of ataxia. Interestingly, cerebellar dysfunction might be associated with cognitive deficits, affective changes (“cerebellar cognitive affective syndrome”), and even psychotic symptoms [11,12]. In our case, the diagnostic criteria for PNS recommended by an international panel of PNS experts were not fulfilled [10]. Following Graus et al. [10], we can postulate a “possible non-classical PNS”, but the low-titer and the transient presence of anti-Yo reactivity argues against this hypothesis. The most probable primary diagnosis for our patient is a drug-induced psychotic disorder, possibly based on an increased vulnerability to short-term drug consumption in the context of the structural and functional cerebral/cerebellar changes depicted in the MRI and FDG-PET, respectively. We speculate that the temporary presence of anti-Yo reactivity might mirror an underlying autoimmune process. However, the role of onconeural antibodies in the pathogenesis of PCD remains unclear because of the predominant intracellular localization of their targets [8,10,13]. Other neurodegenerative disorders with frontoparietal atrophy, such as corticobasal degeneration [14] or with parieto-(occipital) atrophy, such as posterior cortical atrophy [15], are unlikely because of the young age of the patient, her family history, and the otherwise normal FDG-PET scan. Conversely, we found no clinical clue that the cerebellar hypometabolism and atrophy might have been caused by an unrelated disease (e.g., neurodegenerative disorders such as SCA or MSA-C).

### Why this case report? The role of intracellular antineuronal antibodies in psychiatry

Why do we report this case even though we were unable to diagnose paraneoplastic anti-Yo syndrome? One reason is to illustrate the complexity of the clinical work-up in this rapidly evolving field of research. While drug induced psychosis might still be the most likely diagnosis for our patient, we still wonder about the causal role of our findings of transient anti-Yo-antibodies, fronto-parieto-cerebellar atrophy, and the cerebellar PET finding. Do these results indicate that we can stop antipsychotic medication once the antibodies disappeared? Do we have to watch out for future gynecological tumors? One might speculate that a paraneoplastic psychotic syndrome heralded a not-yet detectable gynecological tumor. In this constellation, a paraneoplastic psychotic syndrome could function as a life-saving warning sign. Furthermore, which therapeutic approach is the best in such constellations? Should we stick to symptomatic antipsychotic medication or try immune-modulating interventions? All of these questions are important but unanswered to date.

A growing body of knowledge focuses on the association between psychiatric diseases and autoimmune phenomena, including the detection of antibodies against intracellular neuronal antigens. Remarkably, Laadhar et al. reported confirmed well-characterized antineuronal antibodies in five of 103 psychiatric inpatients (4.9%) who were routinely screened for antineuronal antibodies; they found none in the control group. Aside from three anti-Yo cases (one in schizophrenia, two in bipolar disorder), two schizophrenic patients with anti-Ri reactivity were detected. Interestingly, none of the antibody-positive patients developed a tumor, at least up to a follow-up period of five years [16]. From a pathophysiological point of view, cytotoxic T-cell activity and not autoantibodies is assumed to be responsible for neuronal damage in classical PNS [17]. Therefore, we speculate that misled T-cell responses might be a different pathogenetic mechanism in a subgroup of antineural antibody-associated psychotic snydromes. This view might lead to alternative treatment concepts [16].

In summary, in accordance with the study of Laadhar et al. [16], our case report indicates that reactivity against intracellular neuronal antigens might be associated with psychiatric symptoms without a clinical manifestation of PNS or cancer. Similar results with low-antibody titers are known from anti-N-Methyl-D-aspartate-receptor (NMDAR) antibodies in schizophrenia [18]. However, in a large study investigating the prevalence of anti-NMDAR antibodies, unexpectedly high rates of serum positivity were also found in schizophrenic patients (8.6%), as well as in healthy control subjects (10.8%) [19], a result that challenges the clinical significance of isolated serum antibodies without clinical deficits or signs of a disruption of the blood-brain barrier. As in the case of Hashimoto encephalitis, NMDAR antibodies alone might not be the critical pathophysiological agent but are just a heralding marker of a yet unknown immune process that causes neuronal network dysfunction and psychiatric symptoms as a consequence.

### Conclusion

The currently available data on the prevalence of antibodies against intracellular onconeural antigens and their relevance in psychiatric disorders are sparse. Therefore, case reports are essential to gain an improved understanding of the possible links between structural brain changes, antibody findings, and clinical symptoms. Large, prospective studies that examine the prevalence and possible pathophysiological role of antineuronal antibodies in psychiatry are needed.

### Consent

Written informed consent was obtained from the patient for the publication of this case report and any accompanying images. A copy of the written consent is available for review by the editor of this journal.
